# Efficacy, predictability, and safety of small incision lenticule extraction: 6-months prospective cohort study

**DOI:** 10.1186/1471-2415-14-117

**Published:** 2014-10-03

**Authors:** Jae Ryun Kim, Hyung Bin Hwang, Su Joung Mun, Young Taek Chung, Hyun Seung Kim

**Affiliations:** St. Mary’s Hospital, Department of Ophthalmology and Visual Science, College of Medicine, The Catholic University of Korea, #62 Yeouido-dong, Yeongdeungpo-gu, Seoul, 150-713 Korea; Onnuri Eye Clinic, Jeonju, Korea

**Keywords:** Small incision lenticule extraction, SMILE, Myopia, Incision size

## Abstract

**Background:**

To report our experience with small incision lenticule extraction ("SMILE") for myopia treatment.

**Methods:**

In this prospective clinical study, we evaluated 447 eyes from 224 patients with myopia, with and without astigmatism. We followed the patients for 6 months after SMILE.

**Results:**

The mean (±standard deviation, SD) spherical equivalent was -6.75 ± 1.65 diopters (D) preoperatively and -0.21 ± 0.37 D at 6 months postoperatively. Our data showed that 97.9% of eyes were within ±1.0 D and 86.1% were within ±0.5 D of the intended correction. Furthermore, 79.8% had an uncorrected distance visual acuity (UDVA) of 0 logMAR or less (20/20 or better in the Snellen equivalent) 6 months after surgery. Additionally, 48.5% remained unchanged, 41% gained one line of corrected distance visual acuity (CDVA), 7.2% gained two lines of CDVA, 3.3% lost one line of CDVA, and 0.3% lost two or more lines of CDVA. Age was the only predictor for worsening UDVA at 6 months postoperatively in linear regression analyses (0.07 decrease logMAR per increased 10 years of age; P < 0.05). No predictor showed an association with error in spherical equivalent refraction at 6 months postoperatively.

**Conclusions:**

SMILE is an effective and safe refractive surgery. Age was the only predictor that influenced visual outcome, but its effect appeared clinically insignificant. Faster visual recovery is also expected with improved surgical technique.

## Background

Laser-assisted *in situ* keratomileusis (LASIK) has become the standard technique for treating refractive errors for the past decade because its efficacy and refractive stability have been demonstrated. The procedure enables faster visual rehabilitation so patients can quickly return to normal activities [1, 2]. However, this procedure has side effects including dry eye [3], ectasia [4], and traumatically loosened flaps [5]. Recently, small incision lenticule extraction (SMILE) has been proposed as an alternative to LASIK that could avoid these potential side effects [6]. It has been reported that SMILE resulted in fewer dry-eye symptoms and higher corneal sensitivity [7, 8]. Additionally, this procedure should be able to correct higher levels of myopia because of higher stromal tensile strength after surgery [9].

In the current study, we investigated the efficacy, predictability, and safety of SMILE and compared our results with those from previous studies [6, 10–14]. Moreover, we examined predictors that influenced visual outcome as well as the effects of incision length on visual outcome.

## Methods

The Institutional Review Board/Ethics Committee of Yeouido St. Mary’s Hospital approved this study (SC14RISI0036). It was performed in accordance with the tenets of the Declaration of Helsinki. Written informed consent for participation in the study was obtained from all participants. Inclusion criteria for the study were as follows: spherical myopia up to -12.0 D, myopic astigmatism up to -4.0 D cyl, minimum age of 18 years, corrected distance visual acuity (CDVA) of 20/40 or better (>0.3 logMAR), and minimum calculated postoperative residual stromal bed of 250 μm. In addition, patients had no ocular conditions other than myopia. From May 2012 to November 2013, 447 eyes from 224 patients with myopia (with and without astigmatism) who were treated consecutively with SMILE at the Onnuri Eye Clinic, Jeonju, Korea, fulfilled these criteria. Patients were followed for 6 months.

### Preoperative assessment

Patients underwent preoperative examinations including autokeratometry, autorefractometry, intraocular pressure tonometry (CT-80, Topcon Inc., Japan), pupillometry (Colvard, Oasis Medical, Glendora, CA), corneal tomography, corneal thickness measurements (Galilei, Ziemer Ophthalmic System, Port, Switzerland), measurement of uncorrected (UDVA) and corrected distance visual acuity (CDVA), measurement of manifest and cycloplegic clinical refraction, slit lamp evaluation, and fundoscopy.

### Surgical procedure

A VisuMax 500-kHz femtosecond laser (Carl Zeiss Meditec AG, Jena, Germany) was used for SMILE treatment. The surgery was performed bilaterally and under topical anesthesia using three drops of 0.8% oxybuprocain tetrachloride 2–3 min before surgery. The patient was positioned under the curved contact glass of a femtosecond laser and was asked to fixate on a blinking target. Once appropriate centration was achieved, suction was applied to the contact glass. We used 500 kHz, cut energy index 180 nJ femtosecond laser pulsed, and 4.5-μm spot spacing. First, the back of the intrastromal lenticule was created by photodisruption from the periphery to the center, followed by creation of the lenticule front from the center to the periphery and an incision tunnel located at 11 o/c. The lenticule diameter was 6.5 mm and the cap diameter was 7.5 mm. The incision length was 2.0 to 2.5 mm. The intended cap thickness was 100 to 120 μm. After laser treatment, a thin blunt spatula was inserted through the incision site to break the remaining tissue bridges and loosen the stromal lenticule, which was pulled out using McPherson forceps (GEUDER, GmbH, Heidelberg, Germany; M. Blum design) and removed. After the removal of the lenticule, the stromal pocket was flushed with saline. After surgery, all patients received a topical antibiotic for 5 days and a topical steroid for 2 weeks. Hyaluronic acid lubricating drops were prescribed for at least 2 weeks.

### Outcome measures

The follow-up appointments occurred 1 day, 1 week, 1 month, 3 months, and 6 months after surgery. At each visit, CDVA, UDVA, objected and manifest refractions, keratometry, intraocular pressure, corneal topography, and slit lamp examination were performed. Complications were noted.

### Statistical analyses

Statistical analyses were performed using the SPSS software (ver. 18; SPSS Inc., Chicago, IL, USA). Graphics were generated using Microsoft Excel 2007 (Microsoft Corporation, Redmond, WA, USA). All values are given as the means ± standard deviation. Statistical analyses for visual acuity are based on logMAR units. Bivariate correlation analyses and multiple linear regression analyses were used to evaluate prognostic factors for visual outcomes. The preoperative patients and surgical-related parameters included age, gender, eye side (right/left), corneal curvature, corneal thickness, intraocular pressure, attempted change in spherical equivalent refraction, and degree of astigmatism. Pearson’s χ^2^ analyses were used to compare success rates of early postoperative period visual recovery among groups with different incision lengths. P values less than 0.05 were considered to indicate statistical significance.

## Results

### Study population

This study included 293 eyes from 147 females (65.5%) and 154 eyes from 77 males (34.5%). The mean age was 27.05 ± 6.10 years (range, 18–48 years). Table 1 shows the preoperative patient characteristics. The target refraction was emmetropia (±0.25 D) in all eyes. There was no significant difference between parameters for the right and left eyes.

**Table 1:** **Preoperative patient demographics**

Parameter	Mean ± SD	Range
Manifest sphere(D)	-6.18 ± 1.67	-10.0 to -2.25
Manifest cyliner(D)	-1.14 ± 0.78	-4.0 to 0
Manifest spherical equivalent(D)	-6.75 ± 1.65	-10.50 to -2.25
LogMAR UDVA	1.62 ± 0.25	0.5 to 2.0
LogMAR CDVA	-0.056 ± 0.05	-0.2 to 0.2
Corneal power (D)	44.25 ± 1.49	39.4 to 48.3
Central corneal thickness (um)	523.31 ± 31.73	450 to 625

CDVA corrected distance visual acuity, D diopters, UDVA uncorrected distance visual acuity, D diopters, SD standard deviation.

### Efficacy

**Figure 1:**
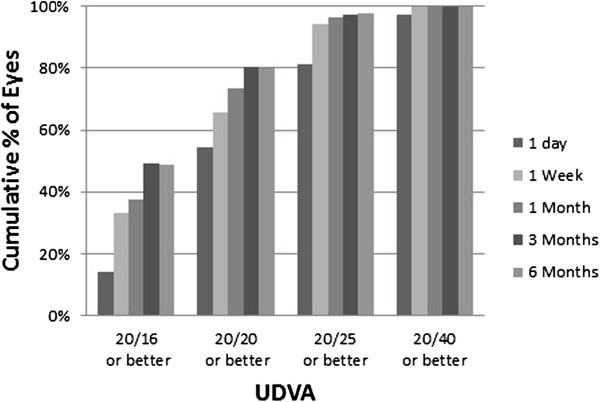
**Efficacy of SMILE.** Cumulative percentage of eyes attaining the specified UDVA levels (Snellen equivalent) at 6 months after surgery.

Figure 1 shows the efficacy of the procedure. UDVA was 20/25 or better in 81.2% of patients 1 day after surgery and 97.6% after 6 months. UDVA was 20/20 or better in 54.4% of patients 1 day after surgery and 79.8% after 6 months. Table 2 shows the patient demographics and rate of visual recovery based on incision length 1 day and 1 week after SMILE. We found that 79.3% of patients in the 2.0-mm group, 79.3% of patients in the 2.2-mm group, 72.2% of patients in the 2.3-mm group, 69.9% of patients in the 2.4-mm group, and 64.7% of patients in the 2.5 mm group had a UDVA of 20/20 or better on the first postoperative day. Additionally, 84.48% of patients in the 2.0-mm group, 81.03% of patients in the 2.2-mm group, 77.78% of patients in the 2.3-mm group, 84.47% of patients in the 2.4-mm group, and 68.54% of patients in the 2.5-mm group had a UDVA of 20/20 or better 1 week after surgery. Patients with smaller incisions had a higher percentage of eyes with 20/20 or better UDVA on the first postoperative day (P < 0.05, Pearson’s χ^2^ test). However, there was no statistically significant difference among the groups at 1 week after surgery (P > 0.05, Pearson’s χ^2^ tests).

**Table 2:** **Patient demographics and SMILE results based on incision length**

Incision length	2.0 mm	2.2 mm	2.3 mm	2.4 mm	2.5 mm	p value
Eyes(n)	58	58	72	103	156	
Age	26.33 ± 6.65	28.52 ± 4.57	26.58 ± 5.21	27.00 ± 6.25	27.03 ± 6.62	0.289
Pre operative SE	-6.95 ± 1.56	-6.59 ± 1.61	-7.01 ± 1.51	-6.65 ± 1.67	-6.68 ± 1.73	0.666
Pre operative CDVA	-0.068 ± 0.038	-0.060 ± 0.054	-0.063 ± 0.059	-0.059 ± 0.056	-0.056 ± 0.051	0.151
% of eyes UDVA						
20/20 or better at 1 day	79.31%	79.31%	72.22%	69.90%	64.74%	0.013*
% of eyes UDVA						
20/20 or better at 1 week	84.48%	81.03%	77.78%	84.47%	68.54%	0.447

SE = Spherical equivalent.

CDVA = corrected distance visual acuity.

UDVA = uncorrected distance visual acuity.

*p<.05.

### Predictability of spherical equivalent (SE)

**Figure 2:**
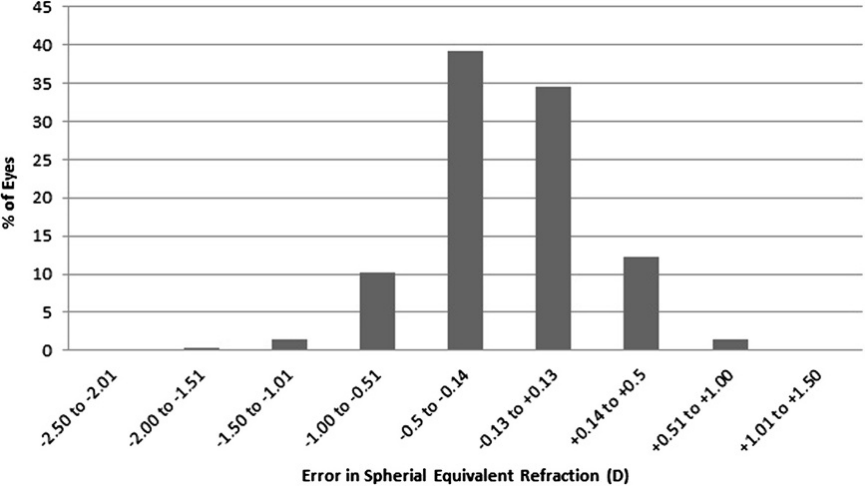
**Predictability of SMILE.** Percentage of eyes within various diopter ranges of the attempted correction at 6 months after SMILE.

**Figure 3:**
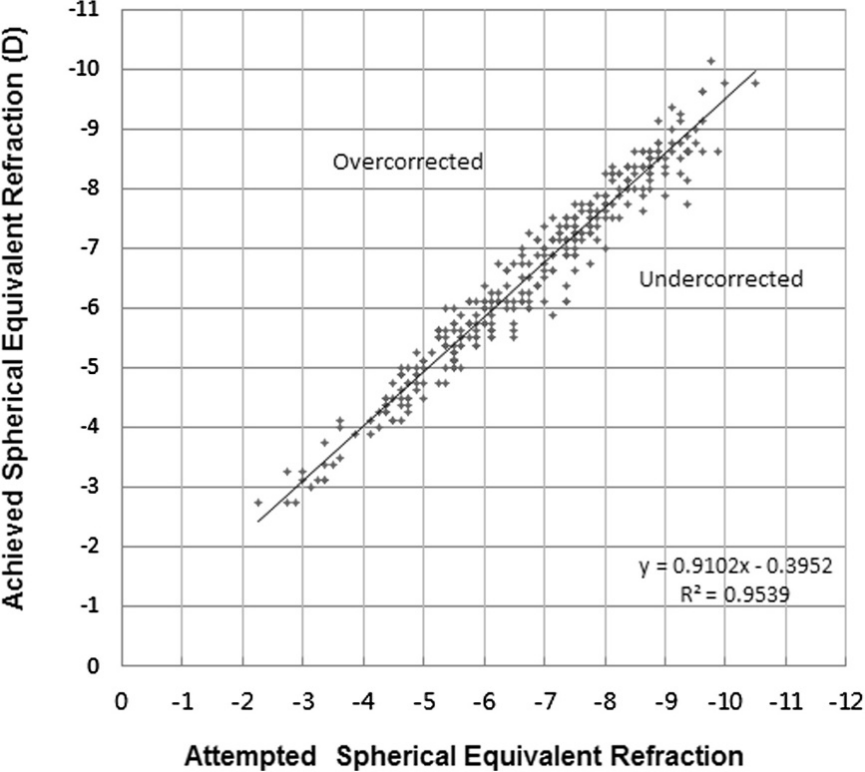
**Predictability of SMILE.** Scatter plot showing attempted versus achieved manifest spherical equivalent (SE) correction at 6 months after SMILE.

Figure 2 shows the predictability at 6 months postoperatively. We found that 86.1% and 97.9% of eyes treated using SMILE were within ±0.5 D and ±1.0 D, respectively, of the intended refractive target after 6 months. Figure 3 shows scatterplot and linear regression analyses of the attempted SE refractive change plotted against the achieved SE refractive change 6 months after surgery.

### Stability and safety

**Figure 4:**
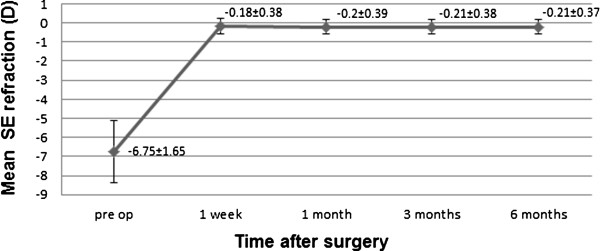
**Stability of SMILE.** Mean SE plotted as a function of postoperative time.

**Figure 5:**
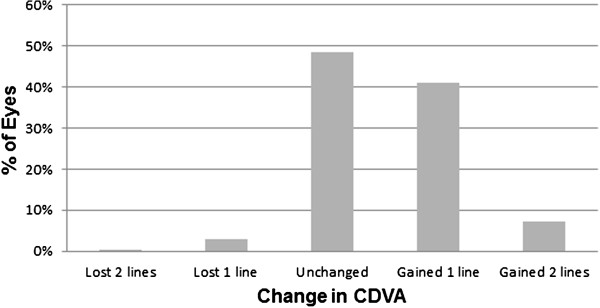
**Safety of SMILE.** CDVA gain and loss at 6 months postoperatively.

Postoperative SE values were -0.18 ± 0.4 D after 1 week, -0.20 ± 0.39D after 1 month, -0.21 ± 0.38D after 3 months, and -0.21 ± 0.37D after 6 months (Figure 4). There was no significant myopic regression from 1 week to 6 months postoperatively (P = 0.355, paired *t*-test). Safety is presented in Figure 5. We report that 48.5% of eyes had an unchanged CDVA, 41% gained one line, and 7.2% of eyes gained two lines. Moreover, 3% lost one line and 0.3% lost two lines of CDVA. No cases of epithelial ingrowth, diffuse lamellar keratitis, or keratoectasia were observed during the follow up period.

### Predictors

Table 3 shows results from bivariate correlation analyses to identify predictors of visual outcome after surgery. According to the analyses, age was the only factor that correlated with UDVA 6 months after surgery. According to linear regression analyses, UDVA worsens as age increases at 6 months after surgery (decrease of 0.07 logMar per decade of age; P < 0.05). However, there were no parameters that correlated with UDVA one day after surgery or with refractive error at 6 months after surgery.

**Table 3:** **Predictors for visual outcome at 6 months postoperatively using bivariate correlation analyses**

Covariate (preoperative factors)	UCVA at 1 day		UCVA at 6 months		Refractive error at 6 months	
	Pearson correlation analysis					
	r	p value	r	p value	r	p value
Age	0.077	0.104	0.139	0.044*	0.106	0.496
Central corneal thickness	-0.017	0.726	0.058	0.293	-0.026	0.639
Corneal power	0.076	0.108	0.006	0.915	-0.01	0.859
Spherical equivalent	-0.035	0.461	-0.162	0.301	0.41	0.081
Astigmatism	-0.051	0.282	-0.087	0.113	0.147	0.347
Intraocular pressure	-0.007	0.88	0.135	0.514	-0.147	0.237

*p<.05.

## Discussion

The present study involved SMILE treatment of moderate-to-high myopia in 447 eyes from 224 patients and included a 6 month follow up period. We controlled for surgical factors (e.g., laser energy setting and treatment zone) to precisely evaluate the predictability, efficacy, and stability of SMILE and to determine predictors that influence visual outcome.

The efficacy, predictability, and safety of SMILE in the present study were promising and comparable with other recently published studies [10–12]. Regarding efficacy, 84%, 73%, and 79% of patients were reported to have 20/25 or better UDVA in various previous studies [10–12]. According to the recent study of Sekundo et al. [13], which reported 1-year results, 98% of patients had 20/25 or better UDVA at 12 months, and 94% and 88% of patients had 20/20 or better at 6 and 12 months, respectively. In the present study, 97% and 79.8% of patients had 20/25 or better UDVA and 20/20 or better respectively at 6 months after surgery. The reason for the lower percentage of 20/20 or better in this study versus that of Sekundo et al. [13] is thought to be the larger number of eyes and higher average degree of myopic correction versus their study.

For predictability, 80.1%, 77%, 91% and 92% were within ±0.5 D and 94.2%, 95%, 98% and 100% were within ±1.0D of the attempted refraction in the various previous studies [10–13]. In the present study, 86.1% and 97.9% of patients were within ±0.5 D and ±1.0 D at the 6-month follow-up.

Regarding safety, Hjortdal et al. [12] reported that 2.4% of eyes lost two or more lines, 15% lost one line, and 83.1% were the same or improved. Anders et al. [11] reported that 4.1% of eyes lost one line, whereas 95.9% were the same or improved after 3 months. In the study by Shah et al. [10], 4% of eyes lost one line, and 96% were the same or improved at 6 months. Similarly, Sekundo et al. [13] reported that 11% of eyes lost one line, and 89% were the same or improved at 12 months.

In the present study, 0.3% of eyes lost two lines, 3% lost one line, and 96.7% were the same or improved at 6 months after surgery. Anders et al. [11] reported that there was a slight but significant regression from 1 week to 1 month after surgery. Sekundo et al. [13] also reported 0.08 D regression after 12 months. In the present study, no myopic regression was observed from 1 week to 6 months. It also needs to be confirmed whether regression will be evident after 12 months of observation.

According to Hjortdal et al. [12], age, corneal power, gender (female), and eye (left) were predictive factors that influenced SE correction errors in multiple regression analyses. These authors suggested that increasing age, corneal power, female gender, and right eye affliction influenced the refractive outcome using an undercorrection. However, in the present study, none of these factors were predictors that influenced errors in SE correction. For predictors that influenced UDVA, Hjortdal et al. [12] reported that increasing age and female gender were risk factors for worsening UDVA at 3 months after surgery. In the present study, however, age was the only risk factor for worsening UDVA at 6 months after surgery (decrease of 0.07 logMar per decade increase in age). However, the effect was so small that it appears clinically insignificant. The present study corroborated the previous predictor analyses of Hjortdal et al. [12] and suggested that SMILE is predictable regardless of diverse patient factors such as gender, corneal power, and amount of correction.

The slight delay in UDVA recovery in the early postoperative period is a unique characteristic of FLEx and SMILE [14–16]. Shah et al. [15] and Demirok et al. [16] reported that initial visual recovery after FLEx was slower than Femto LASIK despite successful refractive correction. Interface haze formation is one of the most common adverse events after this procedure that caused delayed visual recovery in the early postoperative period [17]. However, the incidence of interface haze formation was decreased after using the higher frequency femtosecond laser and applying lower energy, which reduces irregularity of the interface surface [17, 18]. Others have reported that early visual recovery was affected by laser trajectory [15]. A faster visual recovery was noted when the back of lenticule was scanned from the periphery to the center and the front of the lenticule scanned from the center to the periphery. Thus, this preferred method has been used in many studies, including that presented here.

We found that the initial visual recovery was faster compared with recent studies that also used the newer-generation femtosecond laser (500 kHz). Although 40% and 62.3% of patients had UDVA that was 20/25 or better on the first postoperative day in previous reports^11–12^, 81.2% of patients had similar UDVA values in the present study. In particular, 91.4% and 96.6% of patients who received 2.0 mm incision lengths (n = 58) had UDVA 20/25 or better on the first day and 1 week after surgery, respectively.

The faster initial visual recovery noted in the present study may be attributed to the use of different laser settings or to improved surgical factors, such as smaller incision lengths. Laser settings of higher pulse energy and larger spot spacing appear to aid faster initial visual recovery. The laser setting of 180-nJ pulse energy and 4.5-μm spot spacing in the present study showed faster initial visual recovery compared to the previously reported 120 to 150-nJ pulse energy and 2.5-μm spot spacing [11]. Additionally, Hjortdal et al. [12] reported better visual acuity using 170-nJ pulse energy and 4.5-μm spot spacing compared to 125-nJ pulse energy and 2.5-μm spot spacing. Laser settings should be optimized in future studies to improve the initial visual recovery.

To our knowledge, no reported study has evaluated the effect of incision length on visual outcome after SMILE. The present study found that as incision length decreased, visual recovery on the first postoperative day was more rapid (P = 0.013). However, there were no significant differences among the various incision length groups after 1 week. The length of incision did not affect other refractive results, including error in SE and amount of astigmatism after surgery. However, there are several weaknesses in the comparisons among incision groups. Although all eyes were treated by one surgeon, there was no randomization of the incision groups and the numbers of eyes differed significantly by group. Moreover, this analysis of the effects of incision size was, in fact, retrospective, because we had not planned to analyze the effects of incision size on visual outcome when the study was designed. There is also a possibility that the surgeon may have become more skillful when the smaller incision group was treated compared with when the larger incision group was treated, in a relatively early period. In our experience, as the surgeon became adept at smaller incisions, the surgeon could achieve manipulations more readily, as with a larger incision, during the SMILE procedure. Thus, it becomes possible to benefit from the smaller incision, with increased corneal stability and rapid epithelial healing, which may aid in more rapid visual recovery. Presently, we cannot exclude the possibility of better initial visual outcomes because of the smaller incisions, not only greater surgeon experience. The relationship between incision length and HOA after surgery should be evaluated in future studies.

Hjortdal et al. [12] reported that the right eye showed better UDVA on the first postoperative day compared to the left eye. However, we did not observe any difference in UDVA in the left and right eyes 1 day after surgery.

One eye that had undercorrected SE of -1.63 D and 20/40 UDVA after 6 months was retreated in the present study. The eye had a refraction of emmetropia (-0.25 D) and 20/20 UDVA with no other complication 3 months after the surface ablation procedure (PRK).

## Conclusions

The present study had a large number of samples, relatively long-term observation, and treated moderate-to-severe myopia. Our data indicate that SMILE is an effective and safe refractive surgery. Moreover, by developing laser platforms and improving surgical techniques, it is anticipated that the slow visual recovery that is considered a drawback of SMILE can be improved. This procedure would likely be preferred by patients who desire safer refractive surgery with faster visual recovery.

## Abbreviations

SMILE: Small incision lenticule extraction
LASIK: Laser-assisted *in situ* keratomileusis
PRK: Photorefractive keratectomy
UDVA: Uncorrected distance visual acuity
CDVA: Corrected distance visual acuity
SE: Spherical equivalent.

## References

[1] Sandoval HP, de Castro LE, Vroman DT, Solomon KD (2005). Refractive surgery survey 2004. J Cataract Refract Surg.

[2] Ghadhfan F, Al-Rajhi A, Wagoner MD (2007). Laser in situ keratomileusis versus surface ablation: visual outcomes and complications. J Cataract Refract Surg.

[3] Shtein RM (2011). Post-LASIK dry eye. Expet Rev Ophthalmol.

[4] Mohammadpour M, Jabbarvand M (2008). Risk factors for ectasia after LASIK. J Cataract Refract Surg.

[5] Khoueir Z, Haddad NM, Saad A, Chelala E, Warrak E (2013). Traumatic flap dislocation 10 years after LASIK. Case Report Lit Rev J francais d’Ophtalmologie.

[6] Sekundo W, Kunert KS, Blum M (2011). Small incision corneal refractive surgery using the small incision lenticule extraction (SMILE) procedure for the correction of myopia and myopic astigmatism: results of a 6 month prospective study. Br J Ophthalmol.

[7] Li M, Zhao J, Shen Y, Li T, He L, Xu H, Yu Y, Zhou X (2013). Comparison of Dry Eye and corneal sensitivity between small incision lenticule extraction and femtosecond LASIK for myopia. PLoS One.

[8] Li M, Niu L, Qin B, Zhou Z, Ni K, Le Q, Xiang J, Wei A, Ma W, Zhou X (2013). Confocal Comparison of Corneal Reinnervation after Small Incision Lenticule Extraction (SMILE) and Femtosecond Laser In Situ Keratomileusis (FS-LASIK). PLoS One.

[9] Reinstein DZ, Archer TJ, Randleman JB (2013). Mathematical model to compare the relative tensile strength of the cornea after PRK, LASIK, and small incision lenticule extraction. J Refract Surg.

[10] Shah R, Shah S, Sengupta S (2011). Results of small incision lenticule extraction: All-in-one femtosecond laser refractive surgery. J Cataract Refract Surg.

[11] Vestergaard A, Ivarsen AR, Asp S, Hjortdal JO (2012). Small-incision lenticule extraction for moderate to high myopia: predictability, safety, and patient satisfaction. J Cataract Refract Surg.

[12] Hjortdal JO, Vestergaard AH, Ivarsen A, Ragunathan S, Asp S (2012). Predictors for the outcome of small-incision lenticule extraction for Myopia. J Refract Surg.

[13] Sekundo W, Gertnere J, Bertelmann T, Solomatin I (2014). One-year refractive results, contrast sensitivity, high-order aberrations and complications after myopic small-incision lenticule extraction (ReLEx SMILE). Graefe’s Archive Clin Experimental Ophalmol.

[14] Kamiya K, Shimizu K, Igarashi A, Kobashi H (2014). Visual and refractive outcomes of femtosecond lenticule extraction and small-incision lenticule extraction for myopia. Am J Ophthalmol.

[15] Shah R, Shah S (2011). Effect of scanning patterns on the results of femtosecond laser lenticule extraction refractive surgery. J Cataract Refract Surg.

[16] Demirok A, Agca A, Ozgurhan EB, Bozkurt E, Celik U, Demircan A, Guleryuz NB, Cankaya KI, Yilmaz OF (2013). Femtosecond lenticule extraction for correction of myopia: a 6 month follow-up study. Clin Ophthalmol.

[17] Kunert KS, Blum M, Duncker GI, Sietmann R, Heichel J (2011). Surface quality of human corneal lenticules after femtosecond laser surgery for myopia comparing different laser parameters. Graefe’s Archive Clin Experimental Ophthalmol = Albrecht von Graefes Archiv fur klinische und Experimentelle Ophthalmologie.

[18] Blum M, Kunert K, Schroder M, Sekundo W (2010). Femtosecond lenticule extraction for the correction of myopia: preliminary 6-month results. Graefe’s Archive Clin Experimental Ophthalmol = Albrecht von Graefes Archiv fur klinische und Experimentelle Ophthalmologie.

[CR19] The pre-publication history for this paper can be accessed here:http://www.biomedcentral.com/1471-2415/14/117/prepub

